# Putative probiotic lactic acid bacteria isolated from sauerkraut fermentations

**DOI:** 10.1371/journal.pone.0203501

**Published:** 2018-09-07

**Authors:** Tiago Touret, Manuela Oliveira, Teresa Semedo-Lemsaddek

**Affiliations:** CIISA – Centre for Interdisciplinary Research in Animal Health, Faculty of Veterinary Medicine, University of Lisbon, Lisbon, Portugal; Maharshi Dayanand University, INDIA

## Abstract

Probiotics are live microorganisms which confer health benefits to the host, and may be isolated from several sources, such as vegetable foodstuffs. Sauerkraut is a cabbage product resulting from fermentation by a lactic acid bacteria microbial succession, and is a potential source for probiotics. The aim of the present study was the isolation and characterization of probiotic microorganisms from sauerkraut fermentations. Four distinct fermentations were performed, from which lactic acid bacteria were recovered. Overall, 114 isolates were obtained, phenotypically and genotypically characterized, identified to the genus level and evaluated regarding safety and probiotic potential. Representative bacteria were selected for further analysis, 52% being *Lactobacillus* spp. and 33% belonging to *Leuconostoc* spp. genus. One isolate revealed to be β-hemolytic, 42% possessed potentially mobile antimicrobial resistance, 88% were resistant to bile and 20% to low pH. The six most promising candidates were further characterized and presented antimicrobial activity against *Listeria monocytogenes*, three being resistant to lower pH values. Thus, global analysis of data gathered during this study highlighted the identification of three *Lactobacillus* strains with putative probiotic potential, suggesting the applicability of sauerkraut fermentations as a source for probiotic isolation. Due to their origin these strains should be suited for future application in the food industry, namely vegetable products such as sauerkraut itself.

## Introduction

Probiotics are defined as “live microorganisms which when administered in adequate amounts confer a health benefit on the host” [1]. There is an increased interest in probiotics, in face of their recorded safe use and recognized effects on human health [2]. Most probiotics belong to the lactic acid bacteria (LAB) group, which is composed of several genera, including *Lactobacillus*, *Leuconostoc*, *Streptococcus* and *Enterococcus*, among others. These are Gram-positive, oxidase-negative, fastidious and strictly fermentative microorganisms whose main fermentation product is lactic acid [3].

To be selected and applied as probiotics, microorganisms must possess several characteristics. The strain must be safe for human ingestion, with the most important concerns being its virulence potential and the possibility to act as a reservoir for mobile antimicrobial resistance genes [4]. Furthermore, the probiotic candidate must be able to survive the transit through the gastrointestinal tract, in order to maintain viability and potentially colonize the human host [5]. However, colonization may not be necessary for the action of these microorganisms, particularly regarding modulation of the gut microbiota [6]. Finally, the microorganism should provide one or more benefits to human health, with reported beneficial effects including prevention or treatment of acute or antibiotic-associated diarrhea, irritable bowel syndrome, inflammatory bowel disease, necrotizing enterocolitis, and other potential effects including the treatment of obesity [3,7,8]. These diseases are associated with an altered composition of the host microbiota, and one of the proposed mechanism for probiotic effects is through interaction with the microbiota, for example due to competitive exclusion of colonization of pathogenic bacteria. Another possible mechanism is the interaction of probiotics with the host immune system, leading to modulation of the immune response [9].

The two major sources of probiotic microorganisms are the human gastrointestinal tract and fermented dairy products. However, other sources, such as plant based foods, represent important alternatives, since strains isolated from these foods may be more viable and useful for application in similar, non-dairy based probiotic products [2,10].

One possible source of probiotic candidates is sauerkraut, a vegetable product resulting from the spontaneous fermentation of cabbage in anaerobic conditions after the addition of salt [11]. Fermentation occurs over the course of several weeks, at temperatures ranging from 15 to 20°C, and is performed by a microbial succession, characterized by two phases: a heterofermentative phase dominated by *Leuconostoc mesenteroides*, followed by a homofermentative phase dominated by *Lactobacillus plantarum* [12,13]. While these bacteria are the predominant species involved in sauerkraut fermentation, there are other microorganisms present in lower numbers which may be important, mainly other species of *Leuconostoc* and *Lactobacillus*, as well as *Pediococcus* and *Weissella* [13,14]. There have been reports of putative probiotic bacteria isolated from sauerkraut, as well as from related products [15–17], which underlines that it may be a prospective source of probiotic microorganisms.

Due to the relevance of probiotics to human health, it is important to identify bacterial candidates with putative probiotic potential for use in alternative food products. In this context, the main objective of this work was the isolation of LAB from sauerkraut fermentations and the selection of strain(s) with potential to be used as probiotic microorganisms.

## Material and methods

### Sauerkraut fermentations and isolation of lactic acid bacteria

Four sauerkraut fermentations were performed, using either portuguese cabbage (*Brassica oleracea* var. *costata*) or pointed-head cabbage (*Brassica oleracea* var. *capitata*) as substrate. Additionally, two sauerkraut recipes were performed for each type of cabbage, one using just salted cabbage, and the other using this substrate with added aromatic herbs, including lavender, laurel, rosemary, thyme and garlic.

Fermentations were performed as follows: after removing the outer leaves, approximately 300 g of cabbage per assay were washed and sliced into thin strips; then, 3% w/w NaCl (Merck) was added, and the mixture kneaded for a few minutes to allow the release of juices. For the aromatic herbs and garlic recipe, one garlic clove, one laurel leaf and one tablespoon each of lavender, rosemary and thyme were minced and added before kneading. Each mixture was then uniformly distributed in six sterile plastic bottles, filled with a 3% NaCl solution to a final volume of 150 ml and closed tightly. Fermentations were incubated at 20°C for 2, 5, 7, 16, 23 or 30 days. Before starting fermentations (T_0_) and at each time-point, pH was measured and samples were taken from the sauerkraut and fermentation juice for LAB isolation and additional microbial and acid quantification.

Acid production was evaluated by titration of the fermentation juice with NaOH. For this purpose, 5 ml of fermentation juices were diluted in 5 ml of water, and two drops of phenolphthalein were added. This solution was then titrated with 0.01 or 0.1 N NaOH until color change could be observed. The pH of the titrated solution was determined to confirm that the equivalence point had been reached; acidity was calculated as percentage of lactic acid, assuming this was the main acid byproduct.

LAB quantification was performed as follows: 25 g of substrate and 225 ml of buffered peptone water (Oxoid, UK) were added to a stomacher bag, and the mixture was homogenized in a LabBlender 400 stomacher (Seward Limited, UK) for 90 s. Ten-fold serial dilutions were performed in buffered peptone water and inoculated in de Man, Rogosa and Sharpe (MRS) agar (VWR, USA). For the initial salted cabbage samples the 10^−1^ dilution was inoculated, and for the fermented sauerkraut samples the three highest dilutions were chosen, according to results in earlier time-points, and inoculated. Plates were incubated at 37°C for 24–72 h, and colonies were counted in plates presenting 30 to 300 colonies. For each sample, the agar plate with the highest countable number of colonies was selected, and five representative colonies presenting the maximum diversity in terms of morphology were chosen. Colonies were purified by streaking at least four times in MRS agar and conserved in buffered peptone water with 20% glycerol at -80°C, until further use.

### Phenotypic and genotypic characterization of the isolates

Phenotypic characterization was achieved by performing Gram stain, oxidase and catalase tests. Gram-positive, oxidase-negative and catalase-negative (or weakly positive) isolates were submitted to genotypic analysis.

DNA was extracted by the boiling method [18]. Briefly, a colony was suspended in 50 μl of Tris-EDTA with 0.1% Tween 20 (Merck), and incubated for 10 min at 100°C. Then, the bacterial suspension was centrifuged at 14000 rpm for 2 min and the supernatant used directly in PCR reactions.

For fingerprinting analysis, two separate reactions were performed for each of the isolates under study, using two distinct primers. PCR reactions were performed in a final volume of 20 μl, containing 10 μl of NZYTaq 2× Green Master Mix (NZYtech, Portugal), 50 pmol of primer M13 (5’-GAGGGTGGCGGTTCT-3’) or OPC-15 (5’-GACGGATCAG-3’) (StabVida, Portugal) and 1 μl of DNA. Amplification was performed using a Doppio thermocycler (VWR, USA) and the following conditions: 5 min at 95°C; followed by 40 cycles of 1 min at 95°C, 2 min at 40°C and 2 min at 72°C; and a final extension step of 10 min at 72°C, followed by refrigeration at 4°C.

After amplification, 2 μl of GelStar 10X (Lonza Rockland, USA) was added to 10 μl of PCR product and submitted to electrophoresis in a 1.2% agarose gel (NZYtech, Portugal) in 0.5X Tris-Borate-EDTA (TBE) buffer (BioRad). NZYDNA Ladder VIII (NZYtech, Portugal) was used as DNA ladder. Agarose gels were visualized by transillumination under UV light using ImageMaster (Pharmacia Biotech, UK).

Fingerprinting profiles were analyzed using BioNumerics software, version 6.6.5 (Applied Maths, Belgium). Profiles were normalized and grouped using the Pearson correlation coefficient and the unweighted pair group method with arithmetic mean (UPGMA). Dendrograms were produced, and representative LAB chosen for further investigation. The reproducibility level was evaluated by performing 10% randomly selected biological replicates.

Identification at the genus level was performed using a multiplex PCR method specific for *Leuconostoc* and *Lactobacillus* genera, based on genus-specific primers previously described in the literature [19,20]. PCR reactions were performed in a final volume of 20 μl, containing 10 μl of NZYTaq 2× Green Master Mix, 12.5 pmol each of primers LeucA (5’-CACTTTGTCTCCGAAGAG-3’), LeucS (5’-AAGCACTGTTGTATGGGA-3’), LbLMA1-rev (5’-CTCAAAACTAAACAAAGTTTC-3’) and R16-1 (5’-CTTGTACACACCGCCCGTCA-3’) (StabVida, Portugal) and 1 μl of DNA. Amplification was performed using the following conditions: 5 min at 95°C; followed by 35 cycles of 30 s at 95°C, 30 s at 55°C and 1 min at 72°C; and a final extension step of 10 min at 72°C, followed by refrigeration at 4°C. PCR products were submitted to electrophoresis as described above. Presence of a 613 bp amplicon allowed identification of the isolates as *Leuconostoc* sp., whereas a 250 bp amplicon allowed identification as *Lactobacillus* sp. The technique was validated by analyzing relevant reference strains belonging to *Lactobacillus* and *Leuconostoc* genera and other related genera, such as *Pediococcus* and *Lactococcus*. Reproducibility level was evaluated by performing 10% random biological replicates.

Fisher’s exact test was applied to determine if the distribution of *Lactobacillus* and *Leuconostoc* isolates in the fermentations was associated with either the type of cabbage, or the recipe used. Statistical analysis was performed using R, version 3.2.1.

### Safety evaluation

Hemolytic activity was assessed by streaking each isolate in Columbia agar with 5% horse blood (Frilabo, Portugal). Plates were incubated for 24 h at 37°C. Isolates surrounded by a transparent halo were classified as β-hemolytic, and those surrounded by a green halo were classified as α-hemolytic; isolates presenting no halo were classified as γ-hemolytic. *Aeromonas hydrophila* ATCC 7960 was used as a positive control (β-hemolytic), and *Enterococcus faecalis* CECT 795 was used as a negative control (γ-hemolytic).

The antimicrobial resistance profile of the isolates under study was determined using the disc diffusion method, as described by CLSI guidelines [21]. Resistance to ampicillin (10 μg), chloramphenicol (30 μg), clindamycin (2 μg), erythromycin (15 μg), gentamicin (10 μg), kanamycin (30 μg), streptomycin (10 μg) and tetracycline (30 μg) were tested using discs from Oxoid, UK. The mean (M) and standard deviation (SD) of the inhibition halo diameter (x) of all isolates were calculated for each antimicrobial, and isolates were classified as follows: x ≤ M-SD—resistant; M-SD < x < M+SD—intermediate; x ≥ M+SD—susceptible. *L*. *rhamnosus* GG was used as a probiotic control and its results compared with those from existing literature to validate the resistant/susceptible classification [22]. Fisher’s exact test was applied to determine if the antimicrobial resistance of the isolates to each compound was associated with genus allocation. Statistical analysis was performed using R, version 3.2.1.

### Characterization of probiotic potential

Resistance to low pH and bile were tested using a plate assay [23]. Briefly, isolates were grown in MRS broth, overnight at 37°C, and 5 μl of each bacterial culture were spotted in both MRS agar pH 3.5 (adjusted with HCl) and MRS agar supplemented with 0.5% bovine bile (Sigma, USA). Unmodified MRS agar plates were inoculated as control. Plates were incubated for 48 h at 37°C. Visible growth was recorded as positive for resistance, and no growth as negative.

To confirm resistance to low pH, selected LAB were further tested using a microplate assay. Isolates were grown in MRS broth overnight at 37°C. Bacterial cultures were then adjusted to approximately 10^10^ cells/ml using MRS broth, and 2 μl of these cultures were used to inoculate 198 μl of three media: MRS broth, pH 2.5; MRS broth, pH 3.0; and unmodified MRS broth. Each of these assays was performed in triplicate in a microplate, which was then incubated at 37°C. After 3 and 24 h of incubation, 5 μl from each suspension was spotted on MRS agar plates and further incubated at 37°C for 24 h.

Isolates were also investigated regarding their antimicrobial activity against pathogenic bacteria. *Listeria monocytogenes* CECT 935 was used as a pathogenicity indicator. LAB were streaked on MRS agar plates and incubated at 37°C overnight. After incubation, a suspension of *L*. *monocytogenes* was prepared and adjusted to 5.0 McFarland standard. Then, 100 μl of this suspension were used to inoculate 8 ml of molten Brain Heart Infusion (BHI) with 0.7% agar (VWR, USA), and the inoculated BHI medium was overlaid on top of the streaked MRS plates. After solidification, plates were incubated at 37°C overnight, and growth inhibition of the pathogenicity indicator recorded as positive for antimicrobial activity.

Moreover, an agar well diffusion assay [22,24] was performed to verify if this antimicrobial activity was caused by secreted compounds. Briefly, isolates were grown in MRS broth at 37°C, overnight. Cell-free culture supernatants were obtained by centrifugation at 14000 rpm, 4°C, 15 min, followed by filtration using 0.22 μm filters (Merck Millipore, Germany). A suspension of *L*. *monocytogenes* was prepared and adjusted to 0.5 McFarland standard. Then, 250 μl of the bacterial suspension were used to inoculate 25 ml of molten BHI with 1% agar and the inoculated media was plated in empty petri dishes. After solidification, 5 mm wells were produced in the agar, and 50 μl of supernatant were placed in each well. Plates were incubated without inversion at room temperature for 1 h, to allow diffusion of the supernatant, inverted and incubated at 37°C, overnight. Inhibition halos surrounding the wells indicated that the antimicrobial activity against the pathogenic indicator was due to the presence of secreted compounds.

## Results and discussion

### Sauerkraut fermentations

**Fig 1:**
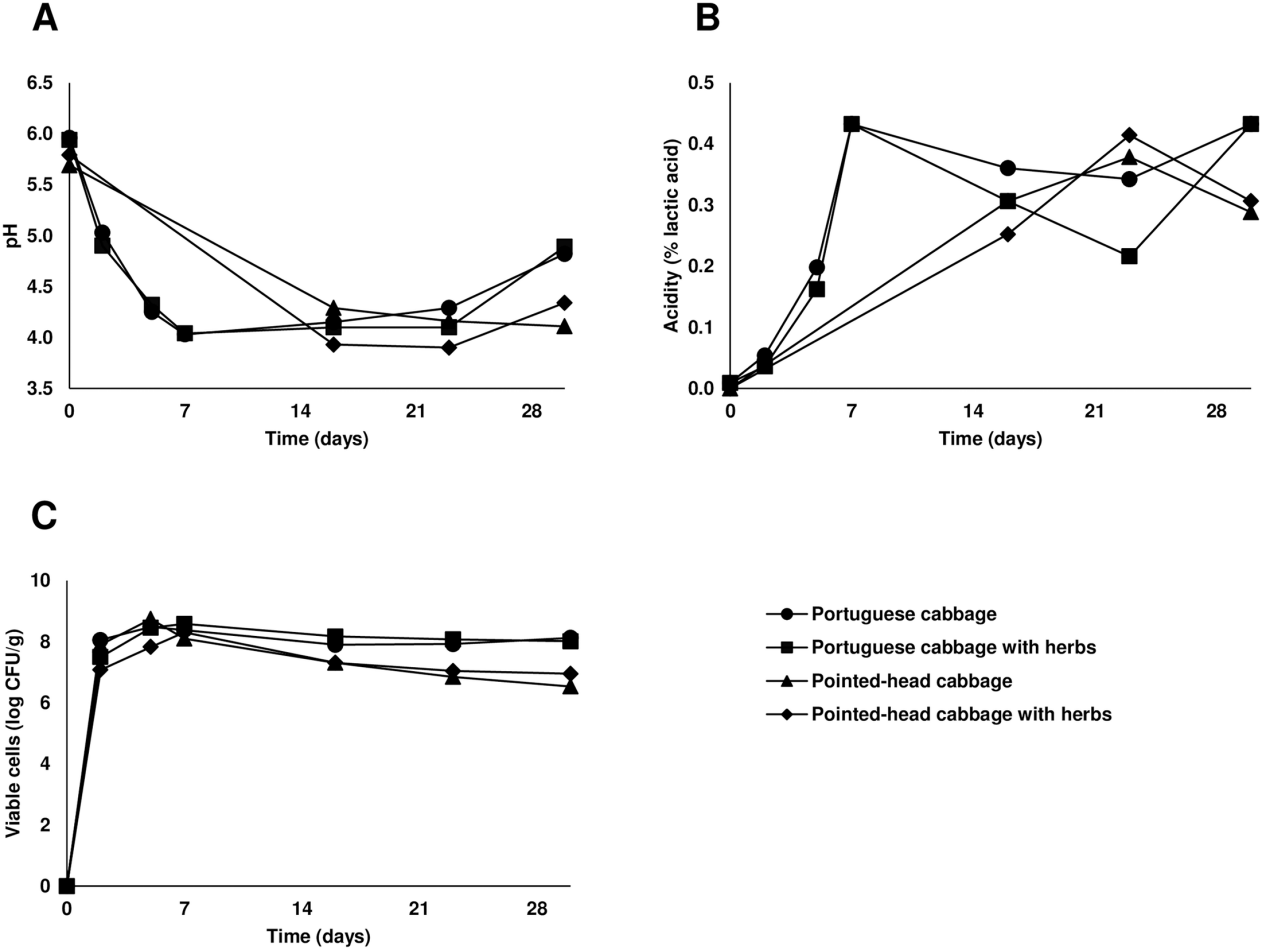
Variation of pH (A), acid content (B) and viable lactic acid bacteria (C) along fermentation time for the four distinct fermentations.

Four distinct sauerkraut fermentations were performed and pH, acidity, and LAB profiles over time were observed (Fig 1). Overall, the majority of chemical and microbiological changes occurred during the first days of fermentation, in particular until day 7. All four fermentations revealed comparable patterns in the measured parameters, with results being most similar in fermentations using the same type of cabbage.

Profiles based on the measured parameters were generally in agreement with those previously described for sauerkraut fermentations, though some differences should be noted: fermentations from the present work reached a lower percentage of acidity (0.3 to 0.4%) and a higher pH value (4.1 to 4.9) than traditionally obtained at similar temperatures (1.6 to 2.3% and 3.5 or less, respectively) [11,25].

This discrepancy can be explained by differences in sauerkraut fermentation conditions, particularly the substrates used. Alternative varieties of cabbage may have a differing composition of nutrients, such as soluble sugars. In fact, it has been described that white cabbage has a higher quantity of sugar than some portuguese cabbage cultivars [26]. The level of this nutrient may be particularly important, since low quantities of sugar in the substrate will lead to a lesser amount of acid production during the fermentation process. However, despite differences observed in the acidity and pH values obtained, the evolution of parameters was similar to previous reports [12].

### Isolation, characterization and identification of lactic acid bacteria

Bacteria were isolated from the four distinct sauerkraut fermentations at selected timepoints (0, 2, 5, 7, 16, 23 or 30 days). A collection of 114 isolates was obtained: from the portuguese cabbage fermentations, 29 (fermentation with herbs) and 35 (fermentation without herbs) LAB were recovered; while from the pointed-head cabbage fermentation, 50 (25 from each recipe) were isolated. Following phenotypic characterization, 95 isolates displaying phenotypic characteristics associated with LAB (Gram-positive, oxidase-negative and catalase-negative/weakly positive) were selected for further characterization.

**Fig 2:**
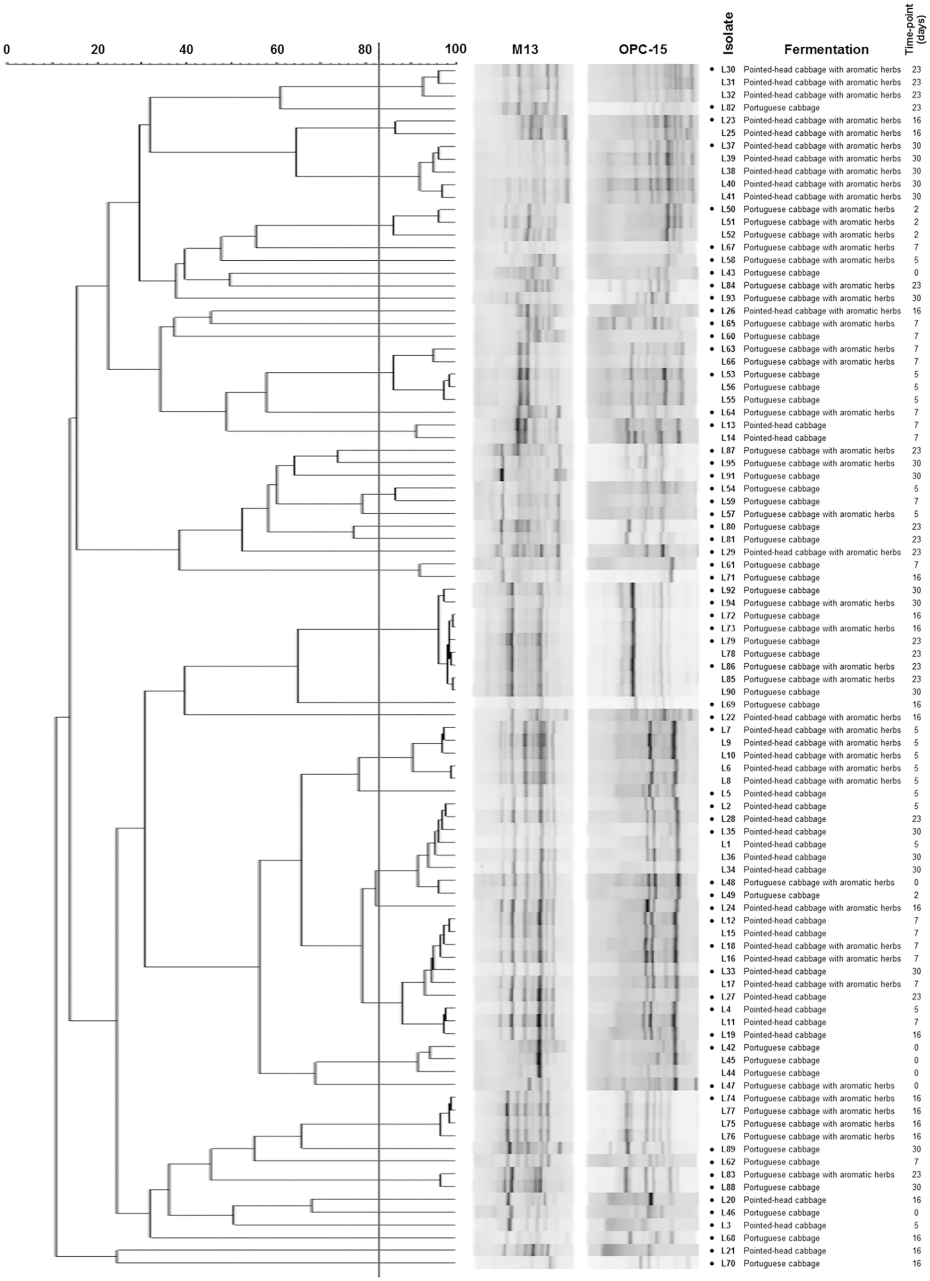
Dendrogram built using M13 and OPC-15 PCR-fingerprinting profiles of the 95 isolates from the four sauerkraut fermentations. The vertical line represents the reproducibility level, which was used as a cut-off value for the definition of genomically distinct LAB. Isolates highlighted with a dot (●) were chosen as representatives for further studies.

Subsequently, to allow the selection of genomically distinct isolates, PCR-fingerprinting was performed (Fig 2). Isolates with similarity above the reproducibility level (83.3%) were considered genomically similar, leading to the selection of 63 representative LAB for each different time-point/fermentation.

Selected LAB were then identified to the genus level by multiplex PCR, using two genus-specific primer sets, targeting *Lactobacillus* and *Leuconostoc* genera. From the 63 isolates, 21 presented a 250 bp amplicon and were identified as *Leuconostoc* sp. (33%), while 33 presented a 613 bp amplicon and were identified as *Lactobacillus* sp. (52%); nine isolates remained unidentified (14%) by this method. A reproducibility of 100% was achieved for this technique.

**Fig 3:**
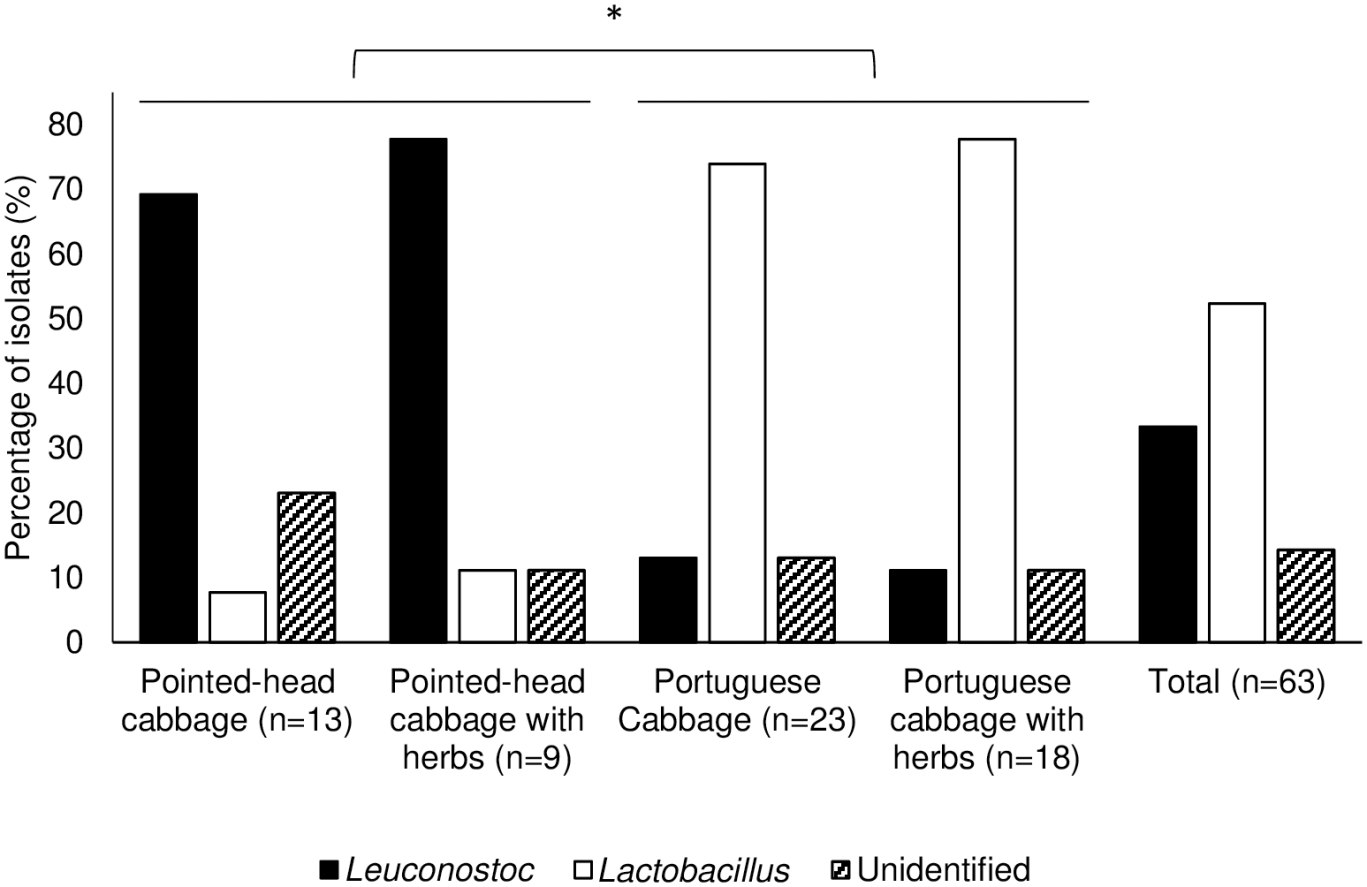
Incidence of *Leuconostoc* and *Lactobacillus* isolates among representatives from the four sauerkraut fermentations. Fisher’s exact test was used to determine statistically significant differences between fermentations. *—P<0.001.

Considering genus allocation and source of isolates, it became evident that distribution of the two genera was not uniform among sauerkraut fermentations (Fig 3). While most isolates in pointed-head cabbage fermentations were identified as *Leuconostoc* (n = 16/22), in portuguese cabbage fermentations the majority belonged to the *Lactobacillus* genus (n = 31/41). Fisher’s exact test was used to compare these two groups of fermentation, showing statistically significant differences (P<0.001). Furthermore, isolates from the fermentations performed with aromatic herbs were also compared to those performed without their addition using the same test, and the results were not statistically different (P>0.05). These results indicate that the distribution of *Lactobacillus* and *Leuconostoc* genera in sauerkraut fermentations is dependent on the type of cabbage used as substrate, but not on the addition of aromatic herbs to the recipe.

**Fig 4:**
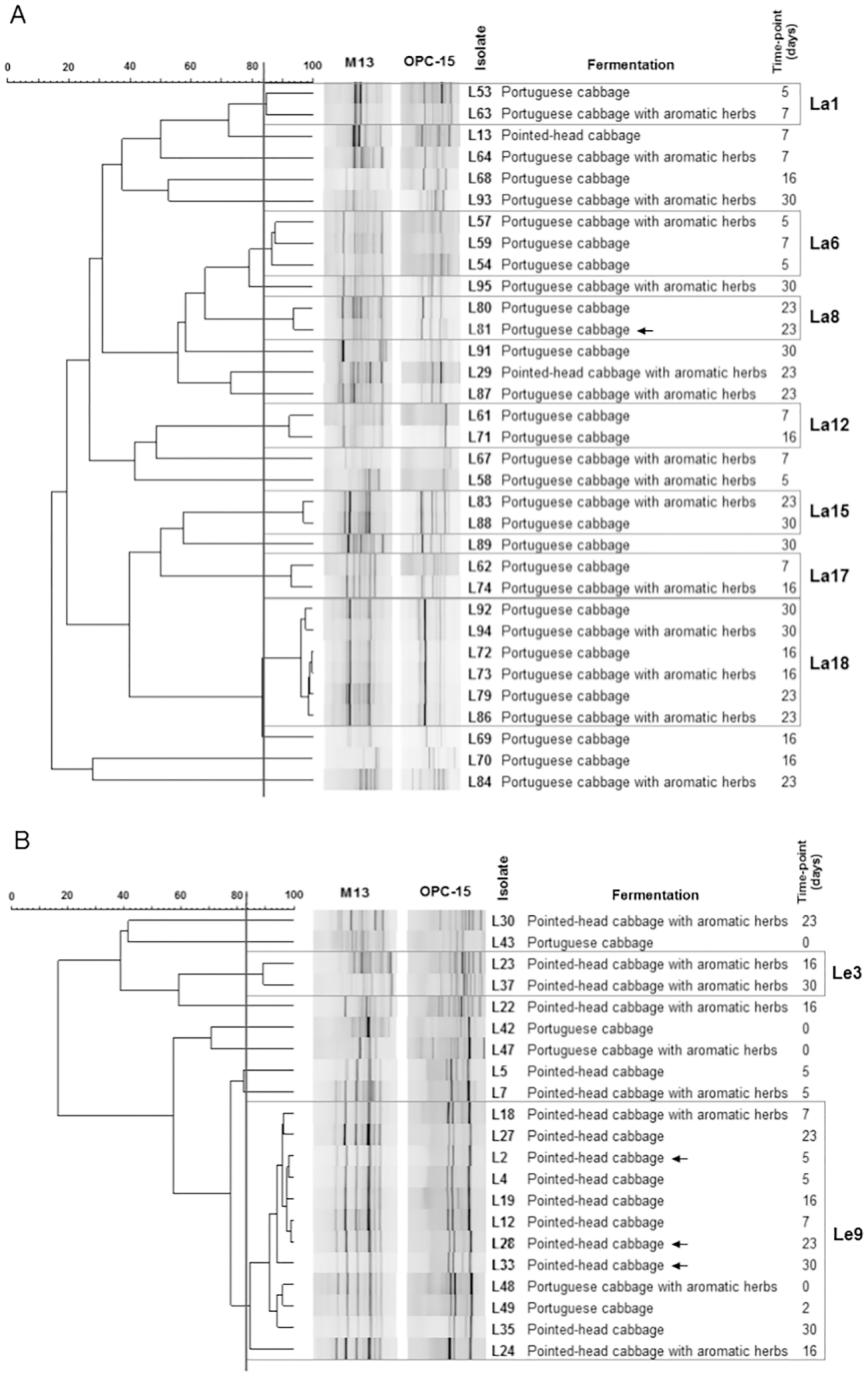
Dendrograms built using the PCR-fingerprinting profiles of *Lactobacillus* (A) or *Leuconostoc* (B) isolates. The vertical line represents the reproducibility level, which was used as a cut-off value for the definition of genomically similar groups. Groups containing more than one isolate are represented. Isolates indicated with an arrow were considered genomically similar to others within the same fermentation/time-point, and were removed from subsequent characterization.

After identification, new dendrograms were created for each genus (Fig 4), and groups of genomically similar bacteria were defined as previously explained. Restricting the analysis to isolates within the same genus clustered four LAB with other isolates of the same fermentation/time-point at high similarity levels, leading to their exclusion from additional studies.

Analysis of groups shared between the fermentations (Table 1) allowed several observations. First, one specific cluster of *Leuconostoc*, Le9, was present at early time-points in every fermentation. Furthermore, microorganisms from this cluster persisted in every time-point in the pointed-head cabbage fermentation without herbs, dominating the fermentation. On the contrary, the other three fermentations showed a more diverse distribution of microbial clusters. Several groups were present in both portuguese cabbage fermentations, with one group of *Lactobacillus*, La18, being present at all time-points from day 16 onwards. This may indicate the importance of microorganisms within this group to the fermentation process.

**Table 1:** Presence of clusters of *Leuconostoc* (Le) and *Lactobacillus* (La) isolates over the course of time, on the four distinct fermentations.

Time (days)	Portuguese cabbage	Portuguese cabbage with herbs	Pointed-head cabbage	Pointed-head cabbage with herbs
**0**	Le2, Le5	Le6, **Le9**	-	-
**2**	**Le9**	-	-	-
**5**	**La1, La6**	**La6**, La14	Le7, **Le9**	Le8
**7**	**La6, La12, La17**	**La1**, La3, La13	**Le9**, La10	**Le9**
**16**	La4, **La12**, **La18**, La19, La20	**La17, La18**	**Le9**	**Le3**, Le4, **Le9**
**23**	La8, **La18**	La11, **La15**, **La18**, La21	**Le9**	Le1, La12
**30**	La9, **La15**, La16, **La18**	La5, La7, **La18**	**Le9**	**Le3**

Groups in bold were present in more than a single time-point, in the same or in distinct fermentations.

Results observed for the portuguese cabbage fermentations are in accordance with the work of Plengvidhya and coworkers [14], which found that most microorganisms isolated until the third day of three sauerkraut fermentations belonged to *Weissella* and *Leuconostoc* genera, while those isolated at the seventh and fourteenth day were from the *Lactobacillus* genus. In the present work, the same distribution was observed in the portuguese cabbage fermentations, with *Leuconostoc* being isolated at the start (T_0_) and second day of fermentation, and *Lactobacillus* from the fifth day onwards.

Results for the pointed-head cabbage fermentations showed a different LAB distribution, with a predominance of *Leuconostoc* spp. at every time-point, which is not usually reported for sauerkraut fermentations. However, a 16S metagenomic study also showed that *Leuconostoc* remained a significant part of the microbiota throughout sauerkraut fermentation [13]. Moreover, Plengvidhya and coworkers [14] observed a different pattern of microbial groups in one of the fermentations studied, with both hetero- and homofermentative species being present at every time-point, which may indicate that distinct patterns of microorganisms can occur in sauerkraut fermentations. Variations found between the various types of fermentations are probably due to the differences in chemical, biochemical and/or microbiological characteristics between the varieties of cabbage used as substrate, affecting the microbial succession. In fact, the different substrates and recipes were used to increase the diversity of LAB and potentially find better probiotic candidates.

### Safety evaluation and assessment of probiotic potential

Hemolytic ability is a relevant virulence factor that can be present in pathogenic microorganisms. Sauerkraut isolates were screened for hemolytic activity (n = 59) and only one was β-hemolytic, with 18 presenting α-hemolysis and 40 showing a γ-hemolytic phenotype. α-hemolytic non-enterococcal LAB have been considered safe by other authors [22,27], suggesting that the majority of the sauerkraut isolates may harbor low virulence potential and could potentially be safe for use as probiotics.

**Fig 5:**
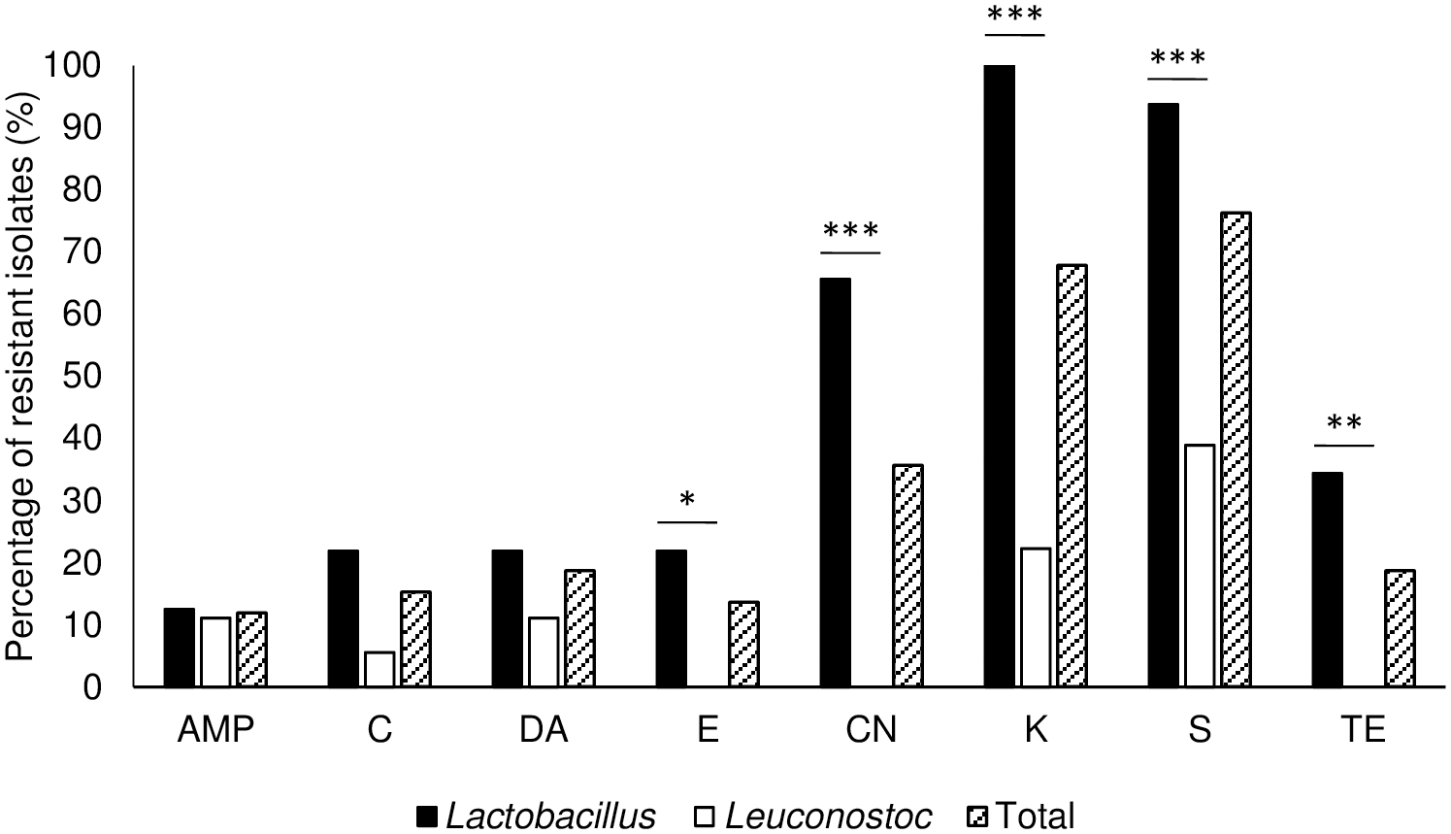
Percentage of isolates resistant to the studied antimicrobial compounds. AMP- Ampicillin; C- Chloramphenicol; DA- Clindamycin; E- Erythromycin; CN- Gentamicin; K- Kanamycin; S- Streptomycin; TE- Tetracycline. Fisher’s exact test was used to determine statistically significant differences between genera. *—P<0.05; **—P<0.005; ***-P<0.0005.

Another important safety concern is the presence of mobile antimicrobial resistance genes. Sauerkraut LAB isolates were assessed for antimicrobial resistance and results are shown in Fig 5. A low percentage of LAB isolates were classified as resistant to ampicillin (12%), chloramphenicol (15%) and clindamycin (19%). For the other antimicrobial compounds tested (erythromycin, gentamicin, kanamycin, streptomycin and tetracycline), statistical analysis showed that the results were genus-dependent (P<0.05). *Lactobacillus rhamnosus* GG, a widely studied probiotic strain, showed resistance to kanamycin.

A high percentage of resistance to gentamicin, kanamycin and streptomycin was detected in *Lactobacillus* spp. These antimicrobials are aminoglycosides, to which lactobacilli have been described as having a high natural resistance [28]. Likewise, the *Leuconostoc* genus is usually reported to be resistant to aminoglycosides [29], but no resistance to gentamicin was found in isolates of this genus in the present work, as observed by other authors [30]. The risk of transmission of aminoglycoside resistance is negligible, so the presence of this characteristic was not applied as criteria for the exclusion of LAB isolates as probiotic candidates.

For the other antimicrobials analyzed, *Lactobacillus* isolates showed a low level of resistance. Resistance to these antimicrobials is not widespread in this genus, although it has been linked to transmission to other microorganisms through genes found in plasmids or transposons [29,31–33], therefore, resistant isolates may act as reservoirs for dissemination. Taking this into account, 42% of the isolates (n = 25/59), found to be resistant to at least one of these antimicrobial compounds, were removed from further characterization.

Study of antimicrobial resistance involves the use of breakpoint values for the classification of microorganisms as resistant or susceptible. Neither Clinical and Laboratory Standards Institute nor EUCAST have defined breakpoints for the study of antimicrobial resistance in *Lactobacillus* or *Leuconostoc* species by disc diffusion [21], for this reason breakpoints were defined for each antimicrobial based on the resistance level of all bacteria included in this study. Isolates presenting an inhibition halo diameter equal or below the mean minus standard deviation of all isolates were considered resistant, while those above this value were considered sensitive or intermediate (non-resistant).

This strategy could lead to a bias in the incidence of resistance, but the observed results were supported by similar findings from LAB isolated from vegetable fermentations, despite the use of different techniques [22,24]. Additionally, the resistance profile obtained for *L*. *rhamnosus* GG was comparable to the observed by Argyri and coworkers [22], indicating that resistance profiles observed in the present study may be comparable to those obtained using different methodologies.

After evaluating the safety of probiotic candidates, isolates were tested for resistance to low pH and bile, important characteristics to survive transit though the human GI tract [5]. For this purpose, an agar-based screening protocol was performed, with results showing that few isolates were resistant to low pH conditions (20%, n = 12/59), all belonging to the *Lactobacillus* genus. Furthermore, a high number of isolates were resistant to bile (88%, n = 52/59). *L*. *rhamnosus* GG was used as a probiotic control and was resistant to both 0.5% bile and a pH value of 3.5.

**Fig 6:**
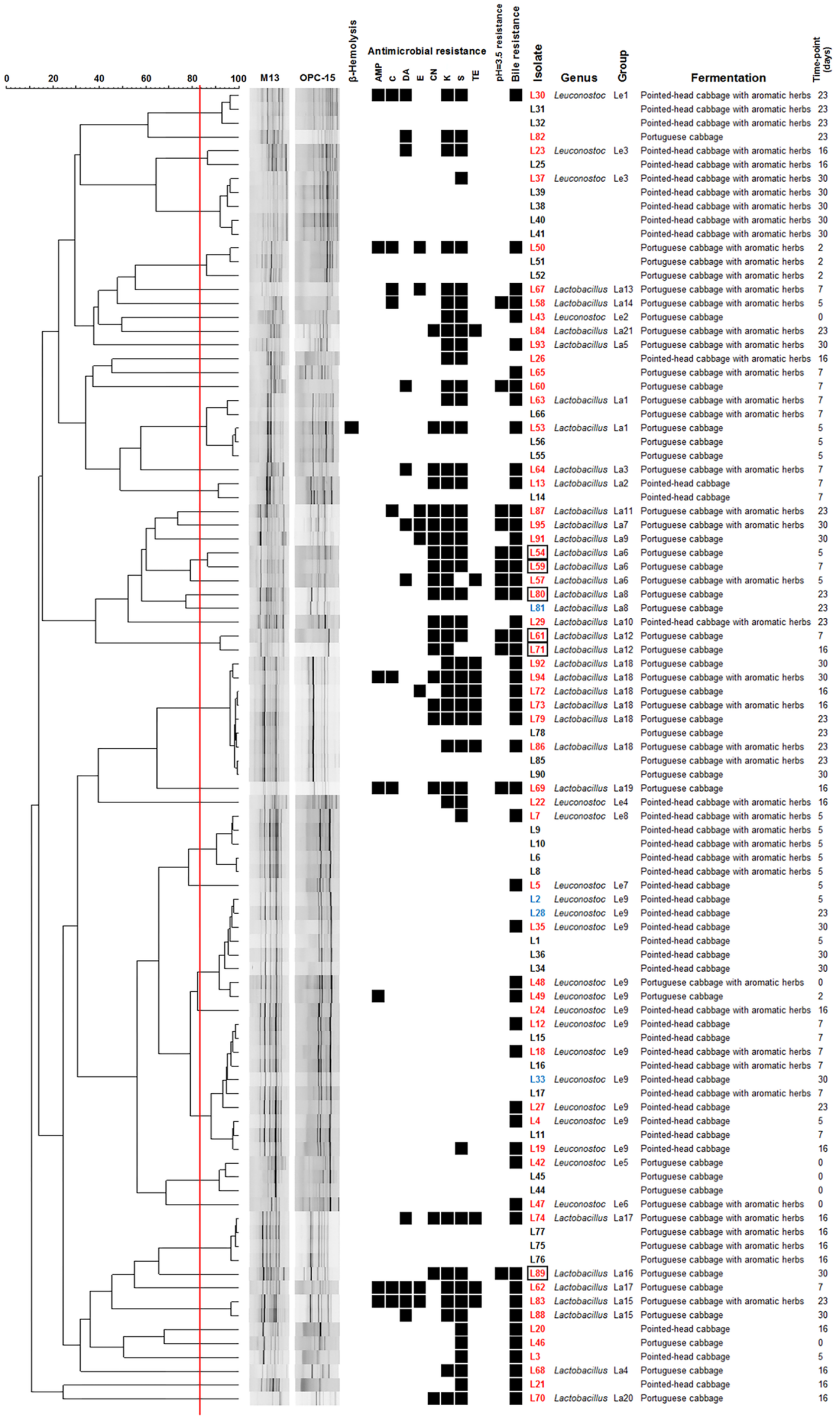
Summary of results for the 95 isolates with a LAB-like phenotype. Dendrogram built based on PCR-fingerprinting profiles, with the red vertical line representing the reproducibility level. Information regarding hemolytic activity, antimicrobial resistance, low pH (3.5) and bile resistance, genus identification, group attributed after PCR-fingerprinting analysis and source of the isolate is also shown. Isolates written in red were chosen as representatives after PCR-fingerprinting, and isolates written in blue were also chosen, but excluded after further analysis. Isolates marked with a box were selected for subsequent analysis based on the information presented in the figure. Black squares represent the presence of AMP—Ampicillin; C—Chloramphenicol; DA—Clindamycin; E—Erythromycin; CN—Gentamicin; K—Kanamycin; S—Streptomycin; or TE—Tetracycline resistance.

Based on hemolytic activity, antimicrobial resistance and resistance to low pH and bile (Fig 6), six *Lactobacillus sp*. (L54, L59, L61, L71, L80 and L89) were selected and tested for antimicrobial activity against *Listeria monocytogenes* and resistance to a lower pH than previously applied. All six isolates were shown to harbor antimicrobial activity against *L*. *monocytogenes* in a spot-on-lawn assay, yet when the inhibitory activity of culture supernatants was tested in an agar well diffusion assay, inhibition was non-existent or very weak. The six isolates were also tested for resistance to lower pH values (pH = 2.5) using a broth-based assay. While viability was observed in four of the isolates after 3 h of incubation, only three (L54, L61 and L89) were still viable after 24 h.

Overall, results showed that probiotic candidates inhibited the growth of *L*. *monocytogenes*, a Gram-positive pathogen representing an important cause of foodborne outbreaks and related mortality [34], but the nature of this effect was not completely established. The antibacterial effect cannot be attributed to soluble compounds present after growth of the isolates in liquid media, such as organic acids or bacteriocins, since these would have inhibited growth in the well diffusion assay. However, antimicrobial activity was observed on the spot-on-lawn test, performed in solid media. A possible explanation is that the production of inhibitory compounds was induced by the presence of the pathogen, since inhibition was only shown when the isolates and the pathogen where in direct contact. In fact, in some cases, co-culture of lactic acid bacteria with target cells can be a requirement for bacteriocin production [35]. Therefore, there is evidence of the antibacterial effect of all the tested isolates against *L*. *monocytogenes*, which further indicates their probiotic potential, although the precise mechanism by which this effect occurred is still not understood.

Results from previous reports regarding acid resistance vary greatly, and this is probably due to the use of different methodologies, which hinder the comparison between results from other studies and the present work. Although there is no established protocol for assessing resistance to low pH, the agar-based methods used in the present study allowed the selection of a small number of isolates with probiotic potential for further testing. The broth-based method allowed to further assess this characteristic, and the fact that three of the six selected isolates were resistant to pH values as low as 2.5 is a good indicator of their suitability as probiotic candidates. Nonetheless, conditions closer to those found in the GI tract, such as the presence of digestive enzymes, should be tested for further confirmation.

Through this work, we were able to identify three lactobacilli showing characteristics associated with probiotics. Isolates belonging to *Leuconostoc* and other genera were also recovered but were excluded as candidate probiotics. In agreement with these findings, Beganović and coleagues [15] isolated strains belonging to these two genera from brines sampled during the course of sauerkraut fermentation of white cabbage, but the two most promising probiotic candidates belonged to the *Lactobacillus* genus. Likewise, Yu and coleagues [17] recovered *Lactobacillus plantarum* from chinese sauerkraut, and identified two potentially probiotic strains. Other studies focusing on kimchi, a fermented vegetable product similar to sauerkraut, showed comparable results [16,36,37].

## Conclusions

The main objective of this study was the isolation of LAB from sauerkraut fermentations and the selection of candidates with putative probiotic potential. Bacteria were isolated from four different sauerkraut fermentations and characterized both phenotypically and genotypically. This allowed the selection of representatives and identification of the majority as *Lactobacillus* spp. or *Leuconostoc* spp. Carefully chosen isolates were then analyzed regarding safety and probiotic features, leading to the selection of three putative probiotic *Lactobacillus* spp. Selection of these strains supports the fact that sauerkraut fermentations are suitable substrates for the isolation of probiotic candidates. Furthermore, these strains may be more appropriate for application in vegetable products, particularly fermented foods such as sauerkraut itself. In fact, due to their origin, they should maintain a high viability in sauerkraut and related products, not leading to a significant change of flavor and other sensory properties, important characteristics for the application of probiotics in non-dairy food products [38].
